# TRPV2 interacts with actin and reorganizes submembranous actin cytoskeleton

**DOI:** 10.1042/BSR20200118

**Published:** 2020-10-14

**Authors:** Manoj Yadav, Chandan Goswami

**Affiliations:** 1 National Institute of Science Education and Research, School of Biological Sciences, Jatni, Khurda 752050, Odisha, India; 2 Homi Bhabha National Institute, Training School Complex, Anushakti Nagar, Mumbai 400094, India

**Keywords:** Actin cytoskeleton, Ca2+-signaling, Growth-cone, Ion channel, Neurites, Neurodegeneration

## Abstract

The understanding of molecules and their role in neurite initiation and/or extension is not only helpful to prevent different neurodegenerative diseases but also can be important in neuronal damage repair. In this work, we explored the role of transient receptor potential vanilloid 2 (TRPV2), a non-selective cation channel in the context of neurite functions. We confirm that functional TRPV2 is endogenously present in F11 cell line, a model system mimicking peripheral neuron. In F11 cells, TRPV2 localizes in specific subcellular regions enriched with filamentous actin, such as in growth cone, filopodia, lamellipodia and in neurites. TRPV2 regulates actin cytoskeleton and also interacts with soluble actin. Ectopic expression of TRPV2-GFP in F11 cell induces more primary and secondary neurites, confirming its role in neurite initiation, extension and branching events. TRPV2-mediated neuritogenesis is dependent on wildtype TRPV2 as cells expressing TRPV2 mutants reveal no neuritogenesis. These findings are relevant to understand the sprouting of new neurites, neuroregeneration and neuronal plasticity at the cellular, subcellular and molecular levels. Such understanding may have further implications in neurodegeneration and peripheral neuropathy.

## Introduction

Regeneration of neurites, especially in the peripheral tissue, has immense importance in the context of different neuronal disorders. The relevance of Ca^2+^-signaling and other signaling pathways in the context of neuritogenesis is well established. However, the molecular mechanisms and the molecular candidates involved in these processes are poorly understood [1–7]. Neuritogenesis is a complex process by which new neurites originate from cell body followed by a series of stochastic events such as neurite extension and/or retraction, bending and often branching at certain points [2,7,8]. Regulation of membrane proteins and membrane dynamics, vesicular recycling, submembranous cytoskeleton and subsequently basic cytoskeletal reorganization are major events involved. All these events are primarily regulated by an array of regulatory proteins that are present on the cell surface, such as ion channels, receptors and adhesion molecules which sense the different chemical signaling cues and allow the neurons to respond accordingly [8–10]. It is well established that inhomogeneous distribution of Ca^2+^-levels is a crucial parameter for most of these processes [2]. Indeed, changes in the spatiotemporal Ca^2+^-levels and Ca^2+^-oscillation patterns have been correlated with most of these functions [2,8–10], which strongly suggest the importance of different Ca^2+^ channels in the regulation of neuritogenesis. Neuritogenesis is critical for proper contact formation, synapse formation and neuronal functions. Therefore, understanding neuritogenesis at cellular and molecular levels are relevant for several neurological disorders including peripheral neuropathy and neurodegeneration.

In this context, previous studies have shown the involvement of Transient Receptor Potential Vanilloid channels (TRPV channels), Ca^2+^ and other signaling molecules to regulate this complex process. The rapid dynamics of neurite extension or retraction in response to chemical cues is well established [11]. Ca^2+^-dynamics and different Ca^2+^ channels are known to regulate neuronal growth cone dynamics [12]. Rapid neurite outgrowth can also be triggered by laser-based micro-heating, suggesting that neurites have unique ability to sense minor temperature differences [13]. Similarly, mechanosensitive Ca^2+^-channels have been implicated in the initiation of neuritogenesis [14]. TRPV channels are members of non-selective cation channels, which have precise thermosensitive and mechanosensitive behavior, and are known to be expressed in peripheral neurons and are involved in several neuronal functions [15–19]. Endogenous expression of different TRPVs in specific neurons and in a specific region of neuronal tissues are highly suggestive of their precise role in neuronal contact formation. Indeed, the importance of TRPV members, particularly TRPV1 and TRPV4 in the context of neurite and synaptic functions have been reported [8,9,20–24]. Other than TRPVs, few other TRP channels and Ca^2+^ channels have also been implicated in such functions. For example, TRPC5 has been implicated in the regulation of neurite movement and growth cone morphology [25]. TRPC channels have been implicated in the netrin-1-induced chemotropic turning of nerve growth cones [26]. Despite all these reports, the full array of Ca^2+^-channels involved in these processes have not been identified and the exact molecular and cellular events involving these channels during neuritogenesis are not well understood.

Based on the existing studies and expression profile, we hypothesize that TRPV2 plays important role in the neuritogenesis process and in different sensory functions. Recently, the involvement of TRPV2 in the regulation of neurite has been demonstrated [10,27]. TRPV2 knockout animals show several abnormalities like cardiac dysfunction and altered immune response [28,29]. Such altered functions can be attributed to the altered neuronal circuit formation and neuro–immune interaction [30–32]. TRPV2 plays an important role during neuronal contact formation and in the process of synaptic plasticity, though the molecular mechanisms involved in such process have not been investigated. Therefore, in this work, we have explored the importance of TRPV2 and its key signaling partners in specific neuronal functions such as neurite initiation, neurite extension and neurite branching.

## Materials and methods

### Reagents and antibodies

Probenecid (P8761) and Tranilast (T0318) were purchased from (Sigma–Aldrich, St, Louis, MO, U.S.A.). All secondary IgG antibodies (Alexa-488-labeled anti-mouse (A-21200), Alexa-488-labeled anti-rabbit (A-11070), Alexa-594-labeled anti-rabbit (A-21442), Alexa-594-labeled anti-rat (A-21472), DAPI (D1306), Alexa-488- labeled Phalloidin (A-12379) and Ca^2+^-sensor dye Fluo4-AM (F14201) were purchased from Invitrogen (Carlsbad, California, U.S.A.). Fluoromount-G (0100-01) was purchased from Southern Biotech (Birmingham, U.S.A.). Anti-TRPV2 antibody (Rabbit ACC-039) for an extracellular loop was purchased from Alomone lab (Jerusalem, Israel).

### Cell culture, transfection and DNA constructs

F11 and CHO K1 cells were cultured in F12 Ham’s medium (HiMedia AL025) supplemented with 10% FBS (HiMedia RM9970), 2 mM l-glutamine (HiMedia TCL012), penicillin–streptomycin (HiMedia A018) (100 units/ml), amphotericin-B (Sigma A2942) 2.5 ng/µl. HaCaT, HEK 293, SaOS and Neuro2a cells were cultured in DMEM (HiMedia) supplemented with 10% FBS (HiMedia, RM9970), 2 mM l-glutamine (HiMedia), penicillin–streptomycin (HiMedia), (100 units/ml), amphotericin-B (Sigma) 2.5 ng/µl. Cells were maintained in a humidified atmosphere at 5% CO_2_ and 37°C. For transient transfection, Lipofectamine 2000 (11668027) and Lipofectamine 3000 (L3000015) (Invitrogen) were used according to the manufacturer’s instructions. For transient expression of TRPV2, fluorescent protein-tagged, plasmid-encoding TRPV2-Wt-GFP, mutant TRPV2-N571T, TRPV2-N572T, TRPV2-NN571-572TT were in pcDNA3.1, and actin-RFP were used [22,33]. Approximately 30 h after transfection, the cells were fixed by PFA (Sigma). GFP-only was expressed by transfecting the cells with pEGFPN3 vector.

### Immunofluorescence analysis

For immunocytochemical analysis, cells were fixed with 4% PFA for 15 min, permeabilized with 0.1% Triton X-100 (T8787) in PBS (5 min) and subsequently blocked with 5% BSA (BSASG100). Primary antibodies raised against TRPV2 were used in 1:500 dilution for 1 h. Alexa 488-conjugated secondary antibody was used at 1:1000 dilution for 30 min.

### Ca^2+^-imaging

F11 cells were cultured on 25-mm glass coverslips. Approximately 24 h after seeding, cells were incubated with Ca^2+^-sensitive dye (Fluo-4 AM, 2 µM for 30 min). Subsequently, coverslips having cells were placed in the live cell chamber and images were acquired. Cells were stimulated with specific agonists or antagonists at the specific frame as described. Fluo-4 AM signal was acquired using NIKON confocal microscope. The images were analyzed using Fiji and intensities for Fluo-4 signal are represented in pseudo rainbow color scale (red and blue indicating the highest and lowest levels of Ca^2+^, respectively).

### Live cell imaging

F11 cells were cultured on 25-mm glass coverslip in complete media. Cells were imaged 36 h after transfection with TRPV2-GFP and/or Actin-RFP. Cells were imaged with ZEISS LSM-780. In some experiments that aimed to evaluate the effect of TRPV2 modulation, certain pharmacological agents were added manually without disturbing the coverslip.

### Image quantification and statistics, software

Images were processed using LSM image examiner software (Zeiss), ImageJ, Fiji and Adobe Photoshop. Quantitative analysis for Fluo-4 was performed by using Fiji. Cellular size and shape were calculated manually for each image using LSM image examiner software (Zeiss). Raw data were imported into GraphPad Prism for statistical analysis. Student’s *t* test was done for two dataset comparison. One-way ANOVA (Dunnett’s multiple comparisons test) was performed for each dataset to get the statistical significance values. Dataset were checked for the normal distribution. The *P*-values were considered as follows: *** <0.001, ** <0.01, * <0.05.

### Protein expression and pull down

Expression and purification of maltose-binding protein (MBP)-TRPV2-Ct (C-terminal cytoplasmic domain of TRPV2 fused with MBP) and as MBP-LacZ (LacZ fused with MBP) was based on a protocol described previously [20]. The expression constructs were introduced into the *Escherichia coli* strain BL21(DE3) by heat-shock method. Fusion protein expression was induced by IPTG (Sigma I5502). Cells were lysed by repeated freeze-thaw cycles. The lysed extracts were cleared by centrifugation and applied to amylose resin. The resins with bound proteins were washed and the proteins were eluted with 10 mM maltose. Approximately 50 µl of amylose resin per tube with the bound MBP-TRPV2-Ct/MBP-LacZ protein was used for pull-down experiments. Soluble brain extract was added to these resin and incubated for 1 h at room temperature (RT) in presence or absence of Ca^2+^ (2 mM). This was followed by three washes with 200 μl PEM-S buffer. The proteins were eluted by 10 mM maltose in 100 µl solution. Eluted samples were analyzed by 10% SDS/PAGE.

### Western blot analysis

After washing in TBST, the membrane was incubated with horseradish peroxidase-conjugated secondary antibody for 1 h at RT (25°C). For the peptide-blocking experiment, equal quantity of cell extract was separated on same gel in side-by-side lanes, the entire gel was transferred to the PVDF membrane (Millipore IPVH00010) as a single blot and then the individual lanes were separated by cutting the membrane into two lanes. After blocking for 1 h in 5% skimmed milk prepared in TBST (20 mM Tris [pH 7.4], 0.9% (w/v) NaCl and 0.1% (v/v) Tween 20), the membranes were incubated with primary antibody for 1 h but in different containers; one piece of membrane with peptide (approximate mole ratio of antibody:peptide is 1:3) and the other one without peptide. Subsequently, both the blots were treated in a similar manner for all washes, secondary antibody incubation and chemo-luminescence detection (according to the manufacturer’s instructions, Thermo Scientific). These membranes were washed in TBST and bands were visualized in chemidoc (Bio-Rad). The exposure of both lanes were for same duration and in same instrument in same settings.

## Results

### Functional TRPV2 is expressed endogenously in F11 cells

**Figure 1:**
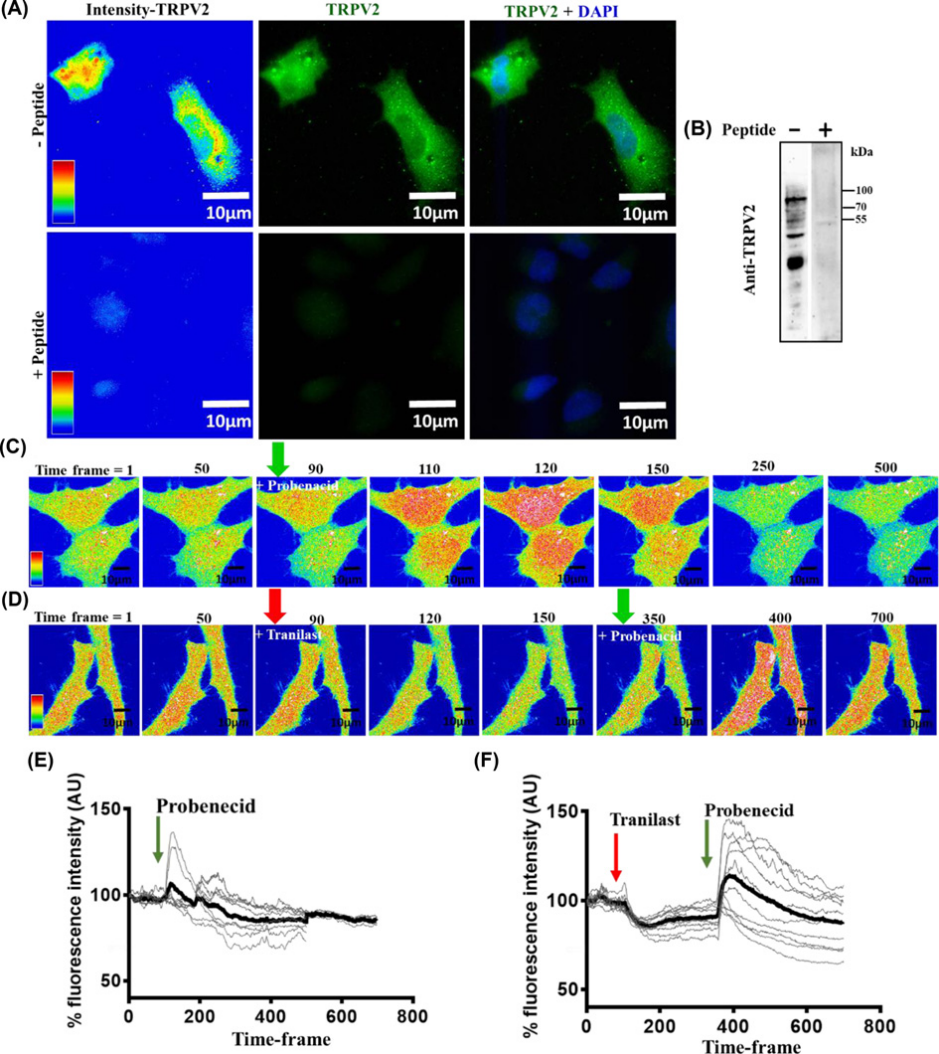
F11 cells endogenously express functional TRPV2 (**A**) Immunofluorescence images of F11 cells stained with anti-TRPV2 antibody in the absence (lower panel) or presence (upper panel) of specific blocking peptides are shown. (**B**) Western blot analysis of F11 cell extract probed with anti-TRPV2 antibody are shown. The presence of specific blocking peptide diminished the TRPV2-specific immunoreactivity completely. (**C**) Live cell imaging of F11 cells incubated with Fluo-4 demonstrating the transient and sharp increase in the intracellular Ca^2+^-level immediately after treating the cells with a specific activator (Probenecid, 250 µM). The interval between each time frame is 0.5 s. (**D**) Similar Ca^2+^-imaging of F11 cells shows an immediate drop in intracellular Ca^2+^- levels followed by application of specific inhibitor (Tranilast, 75 µM). Further application of specific activator (Probenecid, 250 µM) causes a sudden increase in Ca^2+^-level. (**E,F**) Quantification of intracellular Ca^2+^-levels as shown above (C,D) are represented. In each case, fluorescence intensity (in arbitrary units) from multiple cells (*n*=10) are shown. The average value is shown as a thick black line. The interval between each time frame is 0.5 s.

F11 cells are known to endogenously express TRPV2 [37,38]. We investigated the expression of TRPV2 in F11 cells in our culture conditions by immunofluorescence and Western blot analysis. These were performed both in presence and absence of TRPV2-specific blocking peptide (Figure 1A,B). The results confirm the endogenous expression of TRPV2 in F11 cells. In order to confirm this endogenous expression further, we loaded cells with Fluo-4 AM, a Ca^2+^-sensor dye and treated these cells with TRPV2-specific agonists and performed live cell imaging to acquire the changes in the Ca^2+^-level. Activation of TRPV2 by specific agonist (Probenecid) causes a significant increase in the Ca^2+^-level. This rise in Ca^2+^-level is transient in nature and the increased level fades off quickly. Similarly, inhibition of TRPV2 by Tranilast causes a decrease in intracellular Ca^2+^-level. Conversely, further application of Probenecid causes an increase in intracellular Ca^2+^-level (Figure 1C,D). Quantification of the fluorescence intensity using multiple cells confirms our single cell measurements (Figure 1E,F). Taken together, these results suggest that functional TRPV2 is endogenously expressed in F11 cells.

### Overexpression of TRPV2 alters morphology of non-neuronal cells

**Figure 2:**
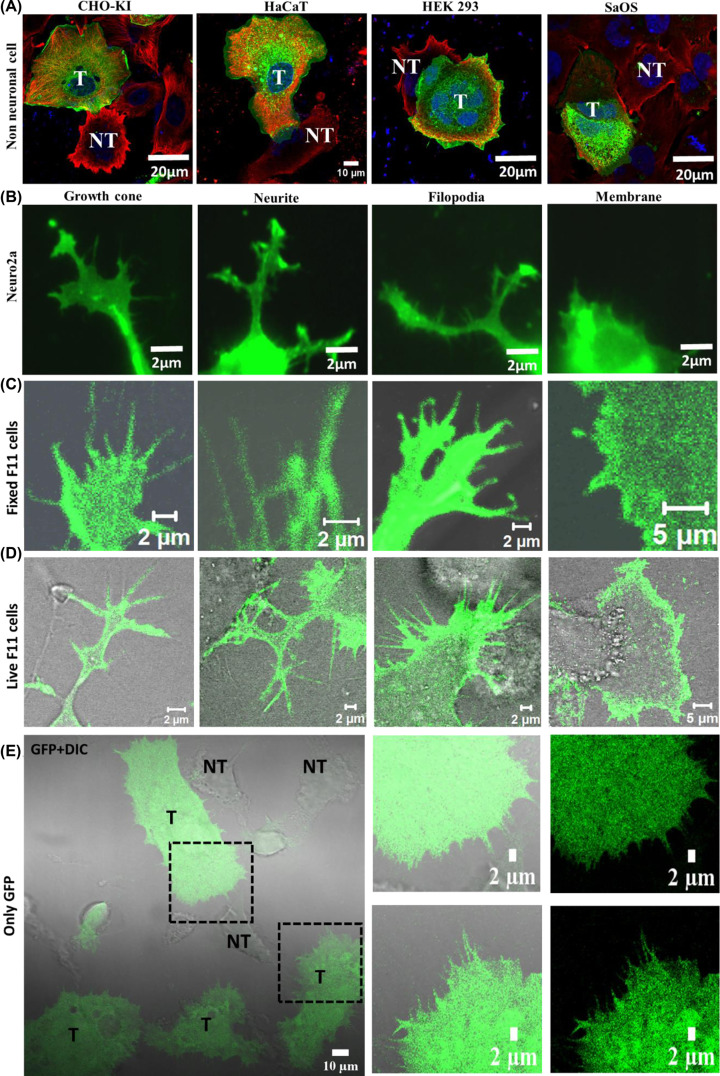
Ectopic expression of TRPV2 alters cell morphology and polarity Confocal microscopy images of different cells and their specialized regions expressing TRPV2-GFP. (**A**) Different non-neuronal cells (CHO-K1, HaCaT, HEK293 and SaOS) become enlarged after expressing TRPV2-GFP, while non-transfected cells retain their normal size (T and NT represent transfected and non-transfected cells, respectively). All cells were stained for Tyrosinated tubulin (Red, YL1/2 Ab) and DNA (Blue, DAPI). (**B**) TRPV2-GFP localizes in specialized cellular structures such as at growth cone, neurite and filopodia when expressed in Neuro2A (neuronal) cells. (**C,D**) TRPV2-GFP localizes in similar specialized cellular structures such as at growth cone, neurite, filopodia in fixed (C) as well as in live (D) F11 (neuronal) cells. In each case, GFP fluorescence is superimposed with the DIC images. (**E**) F11 cells expressing only-GFP do not induce filopodia or neurites. Fluorescence image is superimposed with DIC image. Dotted lines show enlarged areas of the cell.

Though the expression of TRPV2 was initially thought to be restricted to neuronal cells only, later reports confirmed the expression of TRPV2 in different non-neuronal cells. In order to explore the effect of TRPV2 in cell morphology, we expressed TRPV2-GFP in different non-neuronal cell lines like ChoKI (Chinese hamster ovary), HaCaT (keratinocyte cell line), HEK (kidney cell line) and SaOS (primary osteosarcoma). Cells were stained with anti-tyrosinated tubulin-594 and DAPI for better estimation of the morphology of both transfected and non-transfected cells. In all these cases, we noted a drastic change in morphology of TRPV2 expressing cells wherein the cells become much flatter and bigger in size (Figure 2A). Expression of only GFP does not induce such changes (data not shown). This indicates that TRPV2 may play important role in the regulation of submembranous actin cytoskeleton influencing different cellular functions such as cell adhesion and cell spreading.

### TRPV2 localizes in specific membranes, neurites, growth cones and in filopodia of F11 cells

In order to understand the localization of TRPV2 in neurons, we have expressed TRPV2-GFP in Neuro2a cells, and fixed after 48 h. We noted that TRPV2-GFP localizes in specific membranous regions such as in growth cones and in filopodial structures (Figure 2B). Such localizations were also observed in fixed as well as in live F11 cells (Figure 2C,D). Growth cones are specialized structures present at the nerve endings and are involved in neurite extension, neurite bending, cell-to-cell contact formation and in synapse formation [25,34]. Indeed, TRPV2 is also present in the synaptosomes isolated from rat brain (data not shown). Filopodial structures are actin-rich membranous projections and are involved in sensing different environment and chemical stimuli [26,35,36]. The presence of TRPV2 in such membranous regions strongly suggests that TRPV2 might have importance in all these functions and may be involved in complex signaling events regulating actin dynamics and other cellular processes mediated through these structures. To confirm if this effect on change in morphology is indeed mediated by TRPV2, we have used GFP as a control. Cells transfected with only-GFP expressing plasmid do not show any change in morphology and neurite outgrowth (Figure 2E).

### Inhibition but not the activation of TRPV2-GFP results in growth cone retraction and cell retraction

**Figure 3:**
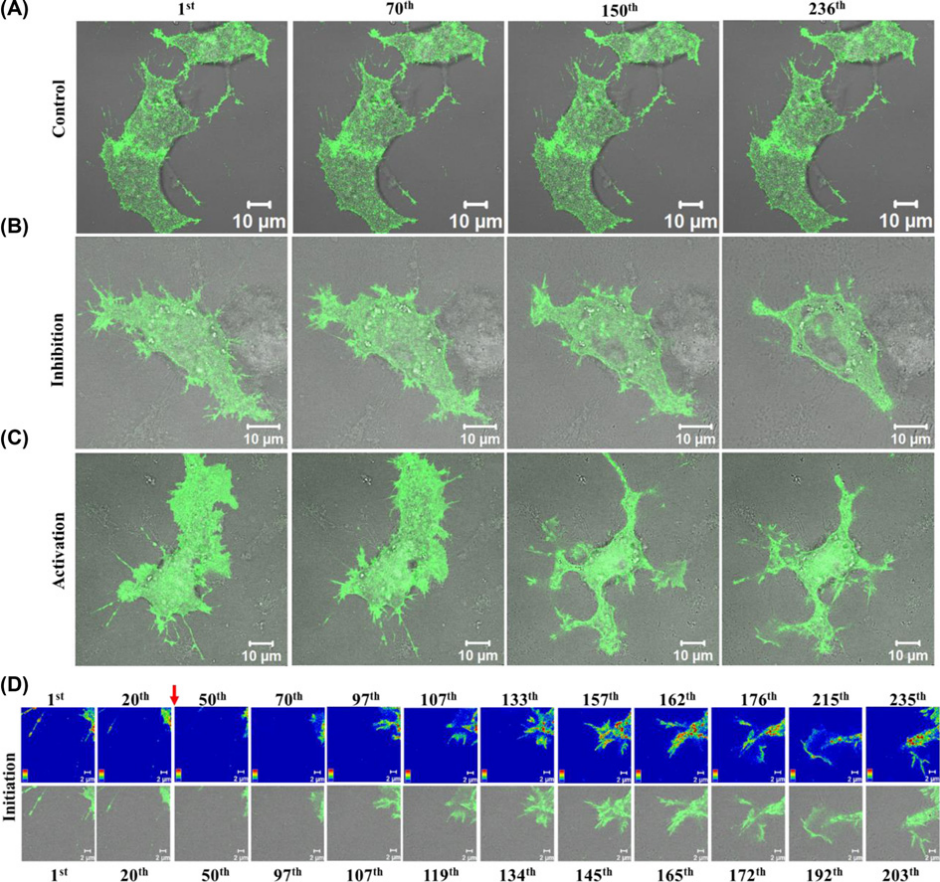
Activation of TRPV2 leads to immediate membrane ruffling and alters filopodial and growth cone dynamics (**A–C**) Representative live cell imaging of F11 cells expressing TRPV2-GFP (green) merged with DIC are shown. When cells were left untreated (A), there is no significant change in the morphology. Inhibition of TRPV2 activity by Tranilast (B) leads to a quick reduction in cell size suggesting that TRPV2 activity contributes to the maintenance of cell morphology. Activation of TRPV2 with Probenecid (C) results in rapid membrane-ruffling leading to changes in cell morphology. In each case, GFP fluorescence is superimposed with DIC. (**D**) Time-series images of enlarged sections of a F11 cell expressing TRPV2-GFP that have been treated with Probenecid (indicated by a red arrow). Activation of TRPV2-GFP results in different events such as initiation of neurites that are controlled by submembranous actin cytoskeleton leading to changes in membrane ruffling. Intensity of the GFP fluorescence is represented in rainbow colors. The time gap between each image frame 0.04 s.

The importance of TRPV1 and TRPV4 in the context of growth cone dynamics is well established [8,22]. Therefore, we tested if TRPV2 acts in the same process. Time-series images from TRPV2-GFP expressing live F11 cells were acquired to monitor the effect of TRPV2 activation or inhibition. For this purpose, cells that express moderate amount of TRPV2-GFP and flattened morphology were used. In resting conditions, these cells do not drastically change their morphology over time (within few minutes of imaging) (Figure 3A). However, pharmacological inhibition of TRPV2 activity by Tranilast results in quick retraction of cells suggesting that TRPV2 activity helps in maintenance of cell size and morphology (Figure 3B). Activation of TRPV2 by Probenecid results in rapid (within few seconds to minute duration) membrane ruffling, changes in lamellipodial and filopodial dynamics (Figure 3C). Such events often result in the initiation of neurites (Figure 3D).

### TRPV2 co-localizes with actin cytoskeleton and interacts with actin through its C-terminal region

**Figure 4:**
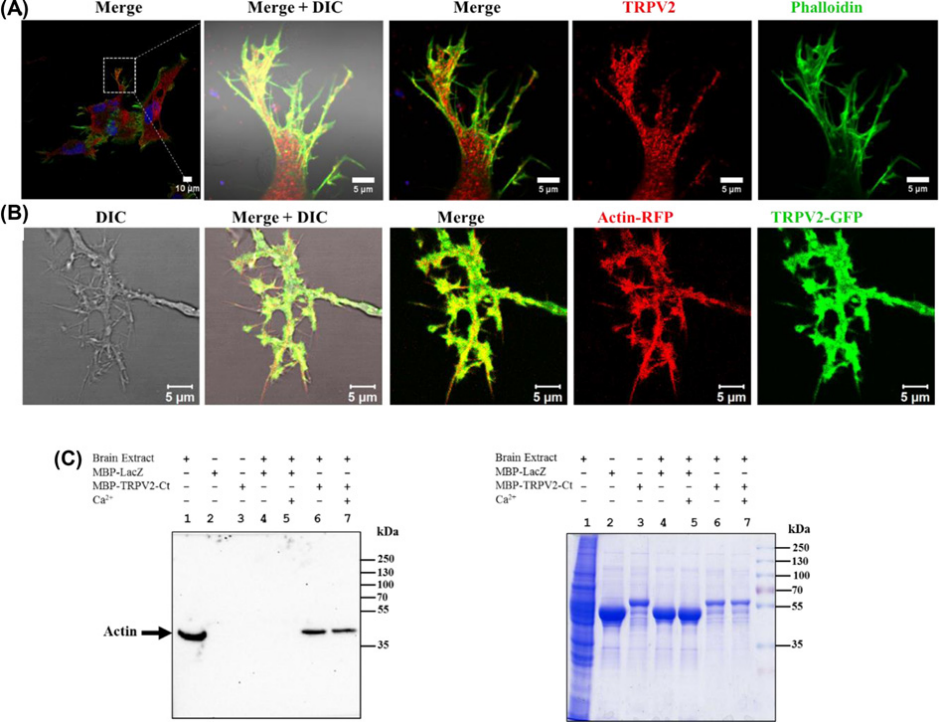
TRPV2 co-localizes with submembranous actin cytoskeleton and interacts with actin (**A**) TRPV2 co-localizes with Phalloidin in filopodia and specific cellular areas. (**B**) Live cell image of F11 cell co-expressing TRPV2-GFP (green) and actin-RFP (red) demonstrating co-localization of TRPV2-GFP and actin-RFP in specific cellular regions such as in neurites and in filopodia. (**C**) The C-terminus of TRPV2 interacts with actin. Soluble brain extract supernatant (lane 1) was pulled down with purified MBP-LacZ (lanes 4–5), MBP-TRPV2-Ct (lanes 6–7) and was probed for bound actin by Western blot analysis.

As TRPV2 is present in typical structures that are enriched with actin cytoskeleton, we explored if both TRPV2 and actin co-localize. For that purpose, we immunostained TRPV2 and labeled actin cytoskeleton by Phalloidin. We noted that TRPV2 co-localizes with actin cytoskeleton in specific regions such as in filopodia and growth cone (Figure 4A). To explore further if TRPV2 co-localizes with actin cytoskeleton in live cell, we co-expressed TRPV2-GFP and actin-RFP and performed live cell imaging. TRPV2-GFP and Actin-RFP co-localize in all these specific cellular areas (Figure 4B).

Next, we tested if TRPV2 interacts with actin. For that purpose, we expressed the C-terminus of TRPV2 as an MBP-tagged protein and performed a pull-down experiment with cattle brain extract. The pull-down samples were probed for the presence of actin by Western blot analysis. Actin interacts with MBP-TRPV2-Ct but not with MBP-LacZ, both in presence and absence of Ca^2+^ (Figure 4C). This confirms that the C-terminus of TRPV2 interacts with actin.

### Activation of TRPV2 causes membrane remodeling

**Figure 5:**
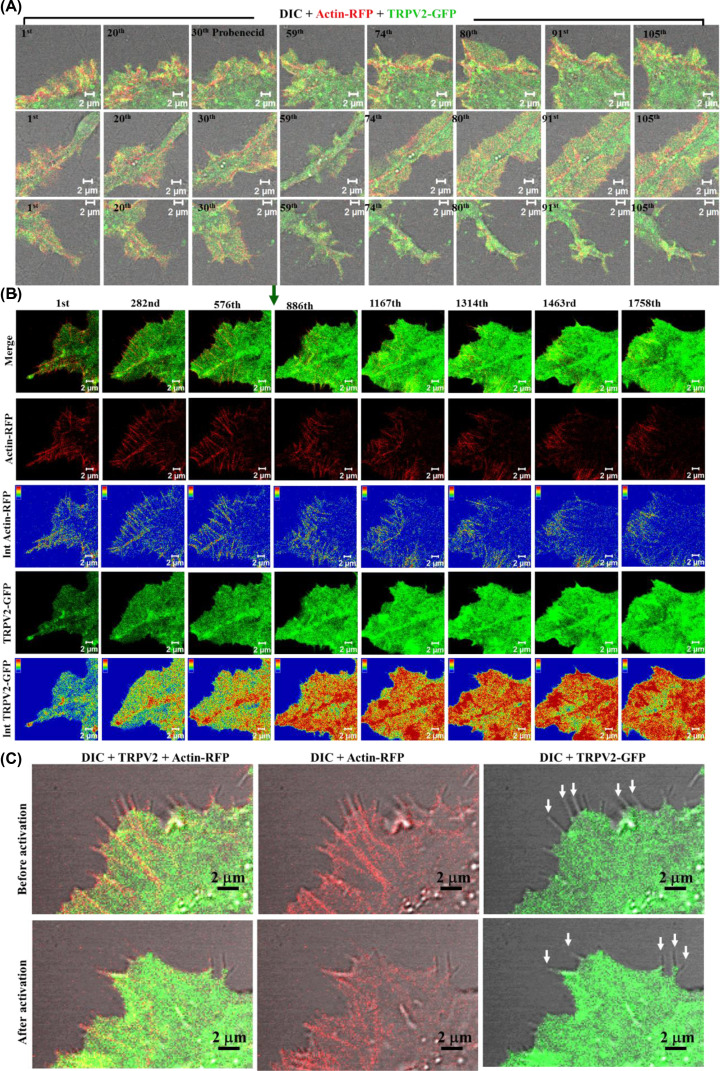
Activation of TRPV2 induces rapid translocation in leading edges but not on the filopodial tips (**A**) Live cell imaging of F11 cell co-transfected with TRPV2-GFP and actin-RFP shows that activation of TRPV2 by Probenecid alters actin cytoskeleton dynamics leading to changes in cell morphology, such as extension of cell membrane (upper panel), formation of massive lamellipodia (middle panel) and merging of lamellipodia (lower panel). (**B**) Activation of TRPV2-GFP by 2APB (indicated by green arrow at 886th frame) also causes changes in submembranous actin cytoskeleton and results in rapid translocation of TRPV2-GFP to the leading edges, merging of actin-ribs in the lamellipodium boundary. Intensity profile of the TRPV2-GFP and actin-RFP are provided below. (**C**) Enlarged section of the leading edge of a F11 cell expressing TRPV2-GFP and actin-RFP before and after activation with 2APB. The filopodial tips are marked with white arrows. TRPV2-GFP is mainly present in the filopodial base but not in the filopodial tips.

Next, we explored if TRPV2 activation and inhibition can cause rapid changes in the cell morphology and if such changes accompany remodeling of submembranous actin cytoskeleton. Activation of TRPV2 by Probenecid leads to rapid changes in actin cytoskeleton causing changes in cell morphology, such as extension of cell membrane, formation of massive lamellipodia and often merging of lamellipodia (Figure 5A). Such effects can also be reproduced by application of 2-aminoethoxydiphenyl borate (2APB; 10 µM), another activator of TRPV2 (Figure 5B). In all cases, such changes are accompanied by rapid translocation of TRPV2 in the membrane, alteration in the actin filaments. Activation of TRPV2 induce rapid translocation in leading edges but not on the filopodial tips (Figure 5C). These results strongly suggest that TRPV2 can be functional for cellular events, such as different aspects of neuritogenesis that involves actin cytoskeleton.

### Exogenous expression of TRPV2 cause changes in cell morphology and induces neuritogenesis

**Figure 6:**
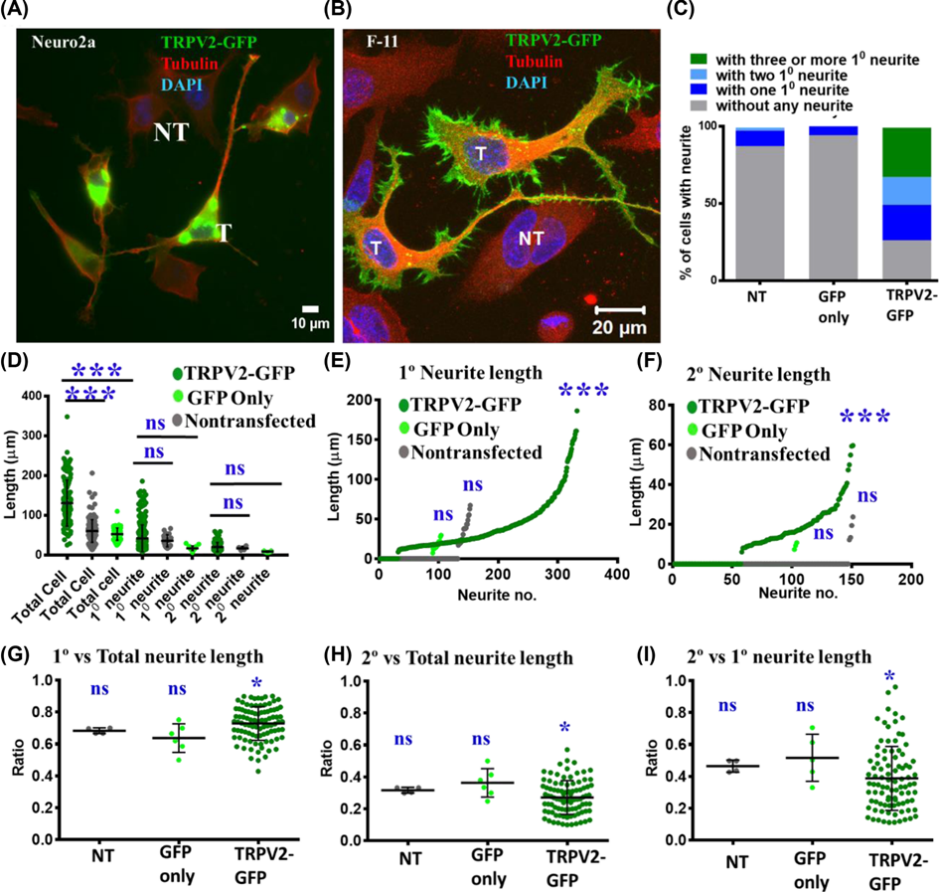
Exogenous expression of TRPV2 induces neuritogenesis and enhances cell elongation (**A,B**) Representative fluorescence microscopic images demonstrating Neuro2A (A) and F11 (B) cells expressing TRPV2-GFP. Transfected cells (T) become much elongated and have a higher number of neurites with complex branches compared with non-transfected (NT) cells. (**C**) Expression of TRPV2-GFP enhances neuritogenesis. Percentage of F11 cells having at least one primary neurite, two primary neurites, more than two primary neurites or no neurites were quantified (*n*=149 for non-transfected cells, 104 for GFP-expressing cells and *n*=131 for TRPV2-GFP expressing cells). (**D**) Quantification of the length of the entire cell (*n*=149), primary (1°) neurite (*n*=100) and secondary (2°) neurites (*n*=80) originating from F11 cells overexpressing TRPV2-GFP are shown. The 131 transfected cells show 301 primary neurites and 94 secondary neurites. Whereas 149 non-transfected cells show 21 primary neurites and 4 secondary neurites. The mean cell length becomes significantly different (*P*-value), while the mean length of the 1° and 2° neurites are non-significantly different. (**E,F**) Length of the 1° and 2° neurites (*n*=131 TRPV2-GFP expressing cells, 104 GFP-expressing cells, and *n*=149 non-transfected cells) present in F11 cells are plotted in ascending orders. For cells without any 1° neurite, the length of the 1° neurite is considered as zero (E). Similarly, for 1° neurite without any 2° neurite, the length of the 2° neurite is considered as zero (F). (**G**–**I**) Ratio (of length) of 1° neurites to total cell (G), 2° neurite to total cell (H) and 1° to 2° neurites (I) are plotted for each cell. Marginal differences in these ratios between TRPV2-GFP expressing cells and GFP-only expressing cells or non-transfected cells are observed. *P*-values ≤0.001, 0.5, 0.1 are considered as ***, * and ns, respectively.

To explore the importance of TRPV2 in functions related to neurite formation and further extension, we transfected TRPV2-GFP in neuronal cells and analyzed the different properties of neurites originated from non-transfected cells as well as cells transiently expressing TRPV2-GFP. Neuro2a cells expressing TRPV2-GFP become much elongated and long neurites are visible (Figure 6A). F11 cells overexpressing TRPV2-GFP become much elongated compared with non-transfected cells and drastic difference in the cell length is visible (Figure 6B). To explore if TRPV2 overexpression can indeed enhance neuritogenesis, we quantified the percentage of cells that show the presence of at least one single primary (1°) neurite, or at least two primary neurites (both originated from the cell body) or even higher number of primary neurites (all originating from the cell body). Approximately 70% of TRPV2-GFP expressing F11 cells develop at least one single neurite within 24 h. In contrast, only ∼25% non-transfected F11 cells develop at least one neurite within the same time points. Similarly, ∼88% cells expressing GFP-only have no primary neurites. The percentage of cells with at least two or more than two primary neurites are much higher in cells that are express TRPV2-GFP than that of the non- transfected F11 cells or GFP-only expressing cells (Figure 6C).

**Figure 7:**
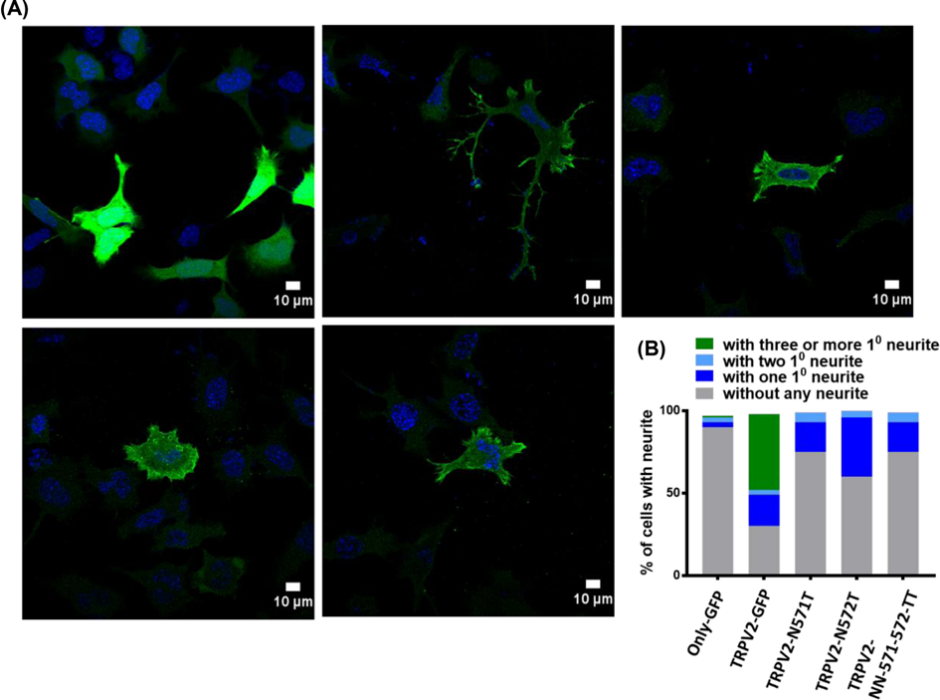
Neuritogenesis is dependent on wildtype TRPV2 (**A**) Only wildtype TRPV2-GFP induces long neurites. The images represent F11 cells expressing TRPV2-Wt-GFP, or TRPV2-mutants in pcDNA3.1 or GFP-only. To find the status of neurites in TRPV2 mutant overexpressing cells were stained with anti-TRPV2 antibody and DAPI and images were acquired by confocal microscopy. (**B**) Quantification of the cells with neurite after expressing GFP only, TRPV2-GFP and TRPV2 mutant are shown. Cells were analyzed by quantifying the cell length without any neurites, with one primary neurite, with two primary neurites and cells with three or more primary neurites. Cells used for the quantification are as follows: GFP-only (104 cells), TRPV2-GFP (26 cells), TRPV2-N571T (49 cells), TRPV2-N572T (25 cells) and TRPV2-NN- 571/572-TT (16 cells) from three independent experiments.

Subsequently, we analyzed the length of the primary and secondary neurites. TRPV2-GFP expressing cells have higher number of primary (1°) as well as secondary (2°) neurites (Figure 6D–F). However, we noted that the average lengths of the 1° or 2° neurites are almost the same (and the differences are non-significant) between cells expressing TRPV2-GFP or that are non-transfected (Figure 6D). Further analysis reveals that the length of the 1° neurites consists majority of the length of the total neurites and the value is marginally more for TRPV2-GFP expressing cells (average value is ∼70%) than non-transfected cells (Figure 6G). The ratio of 2° vs. total neurite length also suggests that 2° neurites contribute ∼25% of the total neurites length and such value is marginally less for TRPV2-GFP expressing cells than that of the non-transfected cells (Figure 6H). Similarly, the ratio of 2° vs. 1° neurites also indicates that the average value is slightly less for TRPV2-GFP expressing cells than that of the non-transfected cells (Figure 6I). In all these cases, expression of only GFP does not induce neurites and these cells are comparable with non-transfected cells only (Figure 7A).

These data strongly suggest that overexpression of TRPV2-GFP induces higher number of neurites per cell (and mainly from the cell body), but does not affect average length of the individual neurites *per se*. Alternatively, these data suggest that overexpression of TRPV2 induces signaling events that cause initiation of more neurites from the cell body, but once the neurites are formed, their lengths are independent of the level of TRPV2 expression *per se*.

### Wildtype TRPV2 induces long neurites

In order to understand the importance of TRPV2 in the neuritogenesis, we expressed wildtype as well as TRPV2 mutants (TRPV2-N571-T, TRPV2-N572-T, TRPV2-NN571-572-TT) which are defective in their properties [33]. To find the mutant overexpressing TRPV2 cells, we stained the cells with anti-TRPV2 antibody. We observed that wildtype TRPV2 and not the mutants induce long neurites (Figure 7A). We have also quantified the cells expressing TRPV2-GFP, only-GFP as a control and different TRPV2 mutants. We have quantified the different parameters such as, how many cells are without any neurites, cells with one primary neurite, cells with two primary neurite and cells with three or more primary neurite. Based on the analysis, we found that majority of TRPV2-GFP expressing cells ∼50% show more than three or more primary neurites. Whereas majority of mutant expressing cells ∼60–80% are without any neurite (Figure 7B). Taken together, the results suggest that TRPV2 promotes the neuritogenesis and neurite branching events.

## Discussion

Regulation of subcellular cytoskeleton and vesicle recycling are essential for several cellular functions, such as cell adhesion, cell spreading, cell–cell contact formation and proper cell signaling events [39–44]. In the case of neuronal cell, specific functions such as filopodial dynamics, growth cone formation, neurite initiation, neurite extension, neurite turning and neurite branching are critical, and relevant for neuronal plasticity [45,46]. Such processes are important for neuron–neuron contact formation and precise neuronal circuit formation which is essential for several physiological and sensory functions [46]. Indeed, such processes are very precise and regulated by multiple factors, and misregulation of such processes often leads to the development of a variety of common pathophysiological conditions. For example, insufficient neuritogenesis may trigger neurodegeneration while excess sprouting may lead to hypersensitivity [47,48].

**Figure 8:**
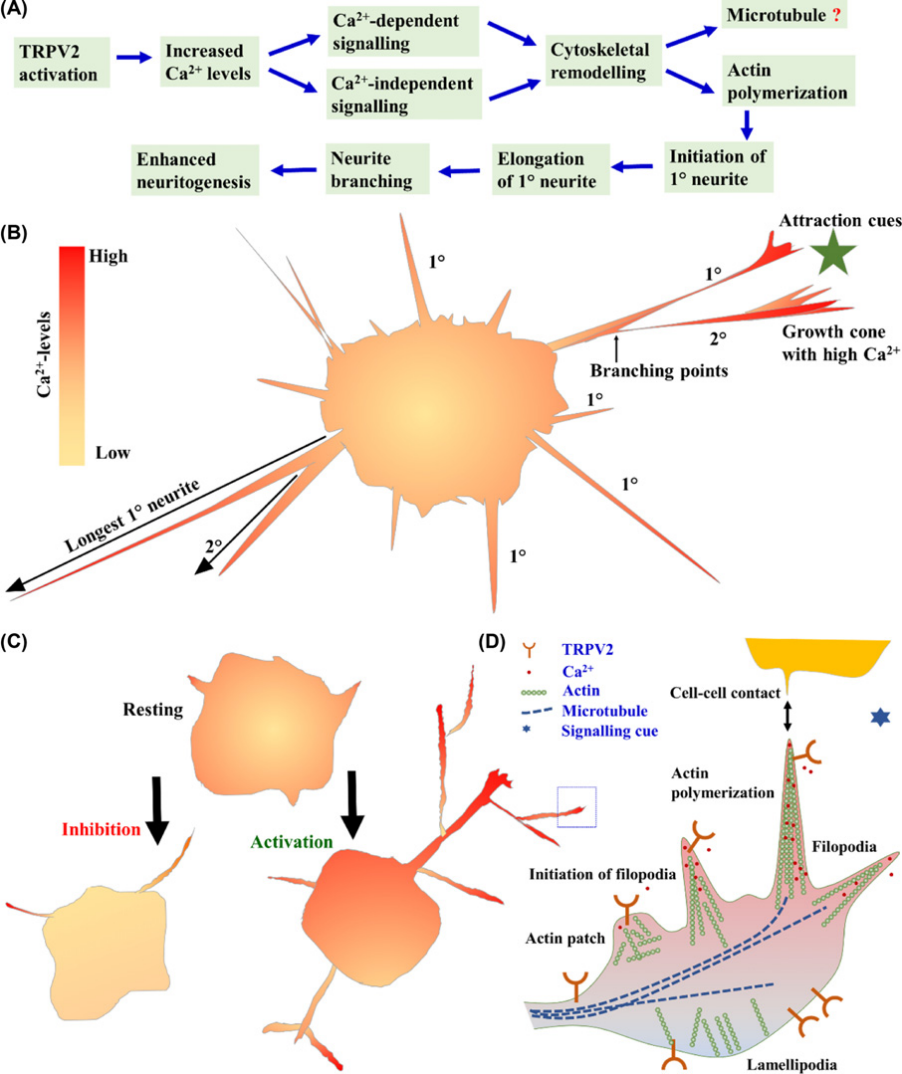
Schematic representation of plausible cellular and molecular events regulated by TRPV2 leading to enhanced neuritogenesis (**A**) TRPV2 activation induces a cascade of cellular events involving both Ca^2+^-dependent and Ca^2+^-independent signaling leading to cytoskeletal remodeling. TRPV2 activation induces new 1° neurites, branching events and induce new 2° neurites leading to enhanced neuritogenesis. However, once initiated, further extension of the formed neurites seems to be independent of TRPV2 activation. (**B**) Schematic representation of a neuronal cell with neurites and their relative intracellular Ca^2+^-levels. TRPV2 seems to contribute to the regulation of Ca^2+^-homeostasis in different parts of the cells and especially in the leading ends where Ca^2+^-levels are usually high. (**C**) Simplified model demonstrating the changes in cell morphology and development of complex neurites due to activation of TRPV2. (**D**) A simplified model represents the involvement of different molecular factors such as TRPV2, actin, microtubule and Ca^2+^ in specialized subcellular structures (indicated by dotted box) such as in filopodia and in neuronal growth cone.

Different ion channels, such as K^+^ and Na^+^ channels are involved in the neuritogenesis events [49,50]. Nevertheless, different steps of neuritogenesis are largely dependent on Ca^2+^-signaling events and thereby involved different Ca^2+^ channels [2,3,7]. Many reports confirmed that different physical and chemical cues significantly affect the process of neuritogenesis [11]. TRPV members are Ca^2+^-permeable channels, polymodal in nature, regulated by different physical as well as chemical cues, external factors and also by endogenous factors. TRPV channels are ideal candidates for sensors that detect different chemical cues, integrate different signals and therefore are functional during neuritogenesis (Figure 8).

Expression of TRPV2 in sensory and motor neurons has been reported in the early stages (E10) of neuronal development [51]. Previously, expression of TRPV2 in specific regions of the brain and in sensory neuron have also been reported [52,53]. In such regions, TRPV2 mainly localizes in neurites and in nerve endings where TRPV2 colocalizes with different synaptic vesicular markers [54]. TRPV2 is also involved in the neuronal contact formation within non-neuronal tissues. For example, of the small calcitonin gene-related peptide (CGRP)^+ve^ neurons projecting to the skin, 11.6% were positive for TRPV2. Involvement of TRPV2 in neuro-protection have also been reported [55]. Here we demonstrate that TRPV2 plays important role in the regulation of neuritogenesis process. Our results are in agreement with previous reports which demonstrate the involvement of other members, namely TRPV1 and TRPV4 in similar functions [8,9,21–23,56].

Our work also accords well with previous works demonstrating the importance of TRPV2 in neuritogenesis in general [10,27]. However, the previous studies were primarily done in PC12 cells, performed in presence of certain growth factors (such as nerve growth factor (NGF) or brain-derived neurotrophic factor (BDNF)), and often TRPV2 was activated by mechanical stimuli or membrane stretching [10,27]. As these growth factors and/or mechanical stimuli can activate a plethora of signaling events, it is difficult to derive the actual involvement of TRPV2 in neuritogenesis from these experiments. Previously, it has been reported that knockdown of TRPV2 by siRNA results in a reduction in NGF-induced neurite outgrowth, at least in *in vitro* culture conditions [10]. However, TRPV2-knockout animals have normal sensory functions suggesting that the neuronal circuits are not altered by the deletion of TRPV2 gene *in vivo* [57]. It is also important to mention that at least one copy of TRPV2 gene is present in all vertebrates suggesting that TRPV2 is essential for overall physiological functions that may not be limited only in the neurons. These facts may also suggest that the role of TRPV2 in neuritogenesis is not essential, and TRPV2 functions are pleiotropic in nature. Nevertheless, the experiments we described in this work are from *in vitro* cell cultures mimicking peripheral neurons and are free from other added growth factors. All the experiments were mainly performed using undisturbed media conditions. Thus, the results described in this work represent the involvement of TRPV2 in neuritogenesis process, specifically in response to pharmacological modulation only and in the absence of any added growth factors or mechanical stimulation-induced activation of TRPV2 (as well as other receptors/channels). So far study of TRPV2 and TRPV3 in neuronal as well as in non-neuronal cells are limited due to the lack of highly specific pharmacological agonist and antagonist that work on exclusively TRPV2 or TRPV3. In this regard, it is worth mentioning that Probeneid, Tranilast and 2APB are fairly specific on TRPV2 and does not have non-specific activation of other ion channels, especially at concentrations used. Our results establish that pharmacological activation of TRPV2 induces neurite initiation and branching. However, once the neurites are initiated, the actual lengths are independent of the TRPV2 expression level. In this context, it is important to mention that the involvement of TRPV2 in the neurite initiation and neurite branching seem to be a complex process that can be enhanced by TRPV2 activation, but certain parameters are unchanged by inhibition of TRPV2, which may involve other signaling pathways.

Filopodial tips responds to mechano-chemical stimuli and micro-heating, which suggest that chemical, temperature and mechano-sensing protein complexes are present [13,58–61]. Because TRPV2 can be activated by temperature, mechanical stimuli and other chemical factors, our observation that TRPV2 is localized in the filopodial structures is in full agreement with the different sensory properties that filopodial structure possesses. Previously, we have reported the presence of other TRPV members, such as TRPV1 and TRPV4 in the filopodial structures. However, the localization and function of TRPV2 in these structures are not exactly like TRPV1 or TRPV4. For example, TRPV1 activation results in retraction of growth cone while activation of TRPV2 does not induce growth cone retraction. In case of TRPV1, it is enriched in the filopodial tips [9,22–24]. In case of TRPV4, rapid translocation of the GFP-tagged channels to the filopodial tips is observed after activation of TRPV4 [22]. However, in case of TRPV2, it is mainly located at the filopodial base and even after activation, it does not translocate to the filopodial tips in general, but mainly remains in the plasma membrane. Interestingly, TRPV2 is known to translocate rapidly to other actin-based structures such as in the nascent phagosome (of macrophages) and podosome where it is involved in the podosome assembly [28,62]. Such differences in localization may indicate the subtle differences of TRP channels associated with different motor proteins present in the filopodia and in the lamellipodia [63]. Nevertheless, we demonstrate that TRPV2 physically interacts with submembranous cytoskeleton, mainly with actin cytoskeleton, which agrees with a previous report showing that the C-terminus of TRPV4 interacts with soluble actin [22]. In this regard, it is important to note that the C-terminus of TRPV2 also interacts with tubulin [33,64]. At present, it is not known if and how TRPV2 regulates cytoskeleton and *vice versa*. Such interactions can be critical for membrane-stretch induced activation of TRPV2. Such interactions might also be important for the regulation of membrane ruffling, membrane spreading, filopodia formation and involvement of different kinase signaling events. However, further experiments are needed to dissect these signaling events in more details.

This work as well as other previous works indicates that TRPV2 is not essential for neuritogenesis, but it can influence the neuritogenesis events significantly and most likely by inducing more branches. Regulation of neuritogenesis and neurite branching are complex process and involvement of different group of molecules such as several receptors, kinases, soluble factors, chemoattractants, Ca^2+^-dynamics and different secondary messengers, microtubule cytoskeleton have been reported. For example, PI3-kinase and NGF-induced signaling events can be relevant for TRPV2-mediated neuritogenesis process [51,65,66]. ERK2-mediated change in Ca^2+^-signaling and NGF signaling are required to initiate the neurite outgrowth [10]. Other factors such as Semaphorin 4D receptor, pituitary adenlylate cyclase-activating peptide (PACAP), BDNF may also be involved in TRPV2-mediated neuritogenesis process, especially in different stages of growth and neuronal connection formation [67–70]. Semaphorin and Plexin interaction has been shown to be crucial to regulate the cytoskeleton protein and involved in the neurite outgrowth and branching [67,68]. Involvement of different neurotransmitters such as Acetylcholine, Dopamine, GABA, Glutamate and Serotonin etc. in TRPV2-mediated neuritogenesis pathways may also be relevant. TRPV2 can be relevant for all these signaling pathways, however these aspects need to be investigated further, also in *in vivo* models. Such findings may help us to understand the cellular and molecular bases of different sensory functions and their implications in different pathophysiological conditions, such as in case of neurodegeneration.

## Abbreviations

2APB: 2-aminoethoxydiphenyl borate
BDNF: brain-derived neurotrophic factor
DAPI: 4′,6-diamidino-2-phenylindole
IPTG: Isopropyl β-D-1-thiogalactopyranoside
MBP: maltose-binding protein
NGF: nerve growth factor
PFA: Paraformaldehyde
TBST: Tris-buffered saline with 0.1% Tween 20
TRP: transient receptor potential
TRPV: transient receptor potential vanilloid
